# Expression of Xenobiotic Metabolizing Enzymes in Different Lung Compartments of Smokers and Nonsmokers

**DOI:** 10.1289/ehp.8861

**Published:** 2006-07-19

**Authors:** Thomas Thum, Veit J. Erpenbeck, Julia Moeller, Jens M. Hohlfeld, Norbert Krug, Jürgen Borlak

**Affiliations:** 1 Drug Research and Medical Biotechnology, Fraunhofer Institute of Toxicology and Experimental Medicine, Hannover, Germany; 2 Bayerische Julius-Maximilians Universität, Medizinische Klinik I, Würzburg, Germany; 3 Immunology/Allergology and Clinical Inhalation, Fraunhofer Institute of Toxicology and Experimental Medicine, Hannover, Germany

**Keywords:** cytochrome P450 monooxygenases, metabolism, smoking, transcription factors, xenobiotic metabolizing enzymes

## Abstract

**Background:**

Cytochrome P450 monooxygenases (CYP) play an important role in the defense against inhaled toxicants, and expression of CYP enzymes may differ among various lung cells and tissue compartments.

**Methods:**

We studied the effects of tobacco smoke in volunteers and investigated gene expression of 19 CYPs and 3 flavin-containing monooxygenases, as well as isoforms of gluthathione *S*-transferases (*GST*) and uridine diphosphate glucuronosyltransferases (*UGT*) and the microsomal epoxide hydrolase (*EPHX1*) in bronchoalveolar lavage cells and bronchial biopsies derived from smokers (*n* = 8) and nonsmokers (*n* = 10). We also investigated gene expression of nuclear transcription factors known to be involved in the regulation of xenobiotic metabolism enzymes.

**Results:**

Gene expression of *CYP1A1, CYP1B1*, *CYP2S1*, *GSTP1*, and *EPHX1* was induced in bronchoalveolar lavage cells of smokers, whereas expression of *CYP2B6/7*, *CYP3A5*, and *UGT2A1* was repressed. In bronchial biopsies of smokers, *CYP1A1*, *CYP1B1, CYP2C9*, *GSTP1*, and *GSTA2* were induced, but *CYP2J2* and *EPHX1* were repressed. Induction of *CYP1A1* and *CYP1B1* transcript abundance resulted in increased activity of the coded enzyme. Finally, expression of the liver X receptor and the glucocorticoid receptor was significantly up-regulated in bronchoalveolar lavage cells of smokers.

**Conclusions:**

We found gene expression of pulmonary xenobiotic metabolizing enzymes and certain key transcription factors to be regulated in bronchoalveolar lavage cells and bronchial biopsies of smokers. The observed changes demonstrate tissue specificity in xenobiotic metabolism, with likely implications for the metabolic activation of procarcinogens to ultimate carcinogens of tobacco smoke.

The lung serves as a primary site for xenobiotic metabolism, and many xenobiotics are substrates for cytochrome P450 catalyzed oxidations. Notably, the lung is composed of > 40 different cell types with different levels of metabolic competence (Ding and Kaminsky 2003). Alveolar macrophages and bronchial epithelial cells are part of > 40 different cell types in lung tissue and differ in their biological functions (Hocking and Golde 1979; Hukkanen et al. 2002). Indeed alveolar macrophages clear the airways of tobacco smoke particles and therefore constitute an important defense mechanism (Drath et al. 1979). Furthermore, the lung epithelium takes part in the detoxification of tobacco smoke through metabolic activation by phase I and phase II enzymes (Crawford et al. 1998; Han et al. 2005). Although a number of CYP isoforms have been identified in human lung tissue, a comprehensive survey of most human pulmonary xenobiotic metabolizing enzymes in different human lung tissue compartments has not been performed.

Tobacco smoke constitutes a complex mixture of thousands of toxicants, some of which require tissue-specific activation to become genotoxic carcinogens and others become metabolically activated poisons (Smith et al. 2003; Yamazaki et al. 1992). For smokers, the risk of developing lung cancer has been shown to be, at least in part, dependent on pulmonary metabolism of smoke constituents (Rubin 2001). Particular evidence stems from studies with smokers in which genetic polymorphisms of certain xenobiotic metabolizing enzymes were linked to the development of lung cancer (Bartsch et al. 2000; London et al. 1999). Currently available data suggest that genetic variability in coding sequences of P450 enzymes, antioxidants, or DNA repair genes contribute to the risk of developing bronchogenic carcinomas (Caporaso 2002).

Cigarette smoke may affect the capacity of lung tissue to dispose of foreign chemicals by changing expression and coded activity of xenobiotic metabolizing enzymes. Additionally, cigarette smoke may affect the pharmacokinetics of inhaled drugs by modulating activity of specific drug metabolizing enzymes (Durak et al. 1996).

Therefore, knowledge of expression and activity of xenobiotic and drug metabolizing enzymes in lung tissue of smokers gives us a better understanding of metabolically induced toxicity of tobacco smoke constituents and allows us to assess the capacity for tissue specific detoxification.

We investigated the gene expression of 19 cytochrome P450 monooxygenases (CYPs) and 3 flavin-containing monooxygenases (FMOs), as well as uridine diphosphate glucuronosyltransferase (UGT) *2A1* (*UGT2A1*), epoxide hydrolase (*EPHX1*), glutathione-*S*-transferase (GST) *A2* (*GSTA2*), *GSTP1*, and *GSTM1* in lung cells obtained from bronchoalveolar lavage (BAL) fluid and in bronchial biopsies (BBs) of smokers (*n* = 8) and nonsmokers (*n* = 10).

Additionally, transcriptional regulation of CYPs depends, at least in part, on promoter activation through binding of transcription factors and nuclear receptors to cognate DNA recognition sites (Borlak and Thum 2001; (Pascussi et al. 2003). We therefore studied expression of the aryl hydrocarbon receptor (*AHR*), constitutive androstane receptor (*CAR*), pregnane X receptor (*PXR*), liver X receptor (*LXR*), and glucocorticoid receptor (*GR*) (Gibson et al. 2002; Tirona et al. 2003) for their established role in *CYP* gene regulation in BAL cells and BBs derived from smokers and nonsmokers.

## Methods

### Study subjects

All subjects were volunteers and gave written consent after being fully informed about the purpose and nature of the study. The study was approved by the Ethical Committee of Hannover Medical School.

Ten nonsmokers and eight smokers were enrolled. All subjects had no history of allergic or other diseases. Only subjects with forced expiratory volume in 1 sec (FEV_1_) > 75% (predicted); normal electrocardiogram, differential blood cell count, blood coagulation, serum parameters (gamma-glutamyl-transferase, aspartate aminotransferase, alanine aminotransferase, urea, creatinine, sodium, potassium, IgE); and negative skin-prick test (ALK-SCHERAX Arzneimittel GmbH, Hamburg, Germany) were included. The characteristics of the study subjects are summarized in Table 1.

None of the subjects had records of drug abuse; for females, pregnancy was excluded before study participation. None of the subjects suffered acute bronchitis 4 weeks before bronchoscopy. Nonsmokers could not have smoked a cigarette for at least 5 years. Inclusion criteria for smokers were a minimum consumption of 15 cigarettes/day for at least 2 years. Levels of cotinine, a stable metabolite of nicotine, were measured in urine to estimate current nicotine exposure. Only smokers with cotinine levels ≥ 100 ng/mL and nonsmokers with cotinine levels ≤ 25 ng/mL were included. In addition, the interval between bronchoscopy and smoking of the last cigarette prior to bronchoscopy was 57–157 min.

### Bronchoscopic procedure

Prior to bronchoscopy, all subjects received atropine (0.5 mg subcutaneously) and midazolam (0.05–0.1 mg/kg). Lidocaine (maximum: 6 mg/kg) was given as a local anesthetic of the upper and lower airways. Drug administration and timing was strictly controlled. The bronchoscope (BF 160 P, Olympus Optical Co. Europe GmbH, Hamburg, Germany) was wedged into the medial segment of the middle lobe, and BAL was performed with 6 × 20 mL sterile saline solution. Lavage fluid from the first 20 mL was discarded. After lavage, the bronchoscope was passed to the anterior segment of the left upper lobe and three bronchial biopsies were taken distal from the carina. The biopsy samples were immediately placed in RNA isolation buffer (Macherey-Nagel GmbH & Co. KG, Düren, Germany), snap-frozen in liquid nitrogen, and stored at −80°C to await further analysis.

### Processing of BAL cells

BAL fluid samples were processed as described previously (Krug et al. 2001). Briefly, cells were filtered through a 100-μm filter. An aliquot of the BAL cells was used to determine the total count of nucleated cells using a Neubauer hemocytometer. After counting, two aliquots containing 1 × 10^6^ cells were separated, pelleted, snap-frozen in liquid nitrogen, and stored at −80°C until RNA isolation. Differential cell counts were obtained from cytospin slides, with 300 cells/slide being counted (Table 2).

### RNA isolation and reverse transcription

RNA was isolated from BAL pellets (1 × 10^6^ cells/pellet) and biopsy materials using the Total RNA Isolation System (Macherey-Nagel GmbH & Co. KG) according to the manufacturer’s recommendation. Quality and quantity of isolated RNA was assayed by capillary electrophoresis (Bioanalyzer 2100; Agilent Technologies Deutschland GmbH, Böblingen, Germany) following the manufacturers instructions. We used 1 μg total RNA from each sample for reverse transcription, as described previously (Thum and Borlak 2002). The resulting cDNA was frozen at −20°C until further experimentation.

### Thermocycler reverse transcriptase-polymerase chain reaction (RT-PCR)

PCR was carried out using a thermal cycler (T3; Biometra GmbH, Goettingen, Germany) as described previously (Thum and Borlak 2001). In brief, the following PCR conditions were used: denaturation, 94°C (45 sec); primer annealing, 54–58°C (60 sec); and extension, 72°C (60 sec). Detailed oligonucleotide sequence information is given in Table 3. We checked for DNA contamination by direct amplification of RNA extracts before conversion to cDNA. Any contamination of RNA extracts with genomic DNA could therefore be excluded. The optimal PCR cycles were derived by studying PCR products at different numbers of PCR cycles. As a consequence, PCR-reactions were performed within the linear range of amplification, and transcript expression levels were calculated as the ratio of the gene of interest (numerator) versus an established housekeeping gene (cyclophilin A, denumerator). We observed no significant differences in the gene expression results when experiments were repeated with the same cDNA, indicating good reproducibility of the method. Amplification products were separated on a 1.5% agarose gel, stained with ethidium bromide, photographed on a transilluminator (Kodak Image Station 440; Kodak GmbH, Stuttgart, Germany), and quantified using the Kodak 1D 3.5 network software.

### *Ethoxyresorufin*-O*-demethylase (EROD) assay*

Immediately after bronchoscopy, approximately 0.1 × 10^6^ cells were separated from BAL fluid and put into 500 μL Dulbecco’s modified Eagle’s medium supplemented with 5% fetal calf serum and 2 mM glutamine. Then, 7-ethoxyresorufin (2 μM, Sigma-Aldrich Chemie GmbH, Deisenhofen, Germany; dissolved in DMSO) and dicumarol (10 μM; Sigma-Aldrich Chemie GmbH) were added, and cells were incubated at 37°C for 4 hr and centrifuged for 5 min (1,200 × *g*, 4°C). The resulting supernatant was snap-frozen in liquid nitrogen and stored at −80°C to await fluorescence detection essentially as described by Grant et al. (1987). Samples (250 μL) were treated with 250 μL ammonium acetate (pH 4.5), and aliquots were treated with 100 U/mL β-glucuronidase (Sigma-Aldrich Chemie GmbH) overnight at 37°C to assay product release of β-glucuronide conjugates. After addition of 500 μL glycine buffer (pH 10.3), fluorometric analysis was carried out on a spectrofluorophotometer (Bio-Rad Laboratories GmbH, München, Germany). The fluorometric analysis of the resultant product resorufin was done with an excitation at 530 nm and an emission wave length of 585 nm. Calibration of the system was performed with resorufin and an appropriate standard curve with a concentration range of up to 100 nM.

### Statistical analysis

We used the Mann-Whitney *U* test for intergroup comparisons. A *p*-value < 0.05 was considered statistically significant. Unless otherwise stated, the data are expressed as median and range or as individual results.

## Results

### Subject characteristics and BAL data

The clinical characteristics of the study subjects are given in Table 1. There was no significant difference in FEV_1_ (% predicted) and FEV_1_/forced vital capacity (FVC) between smokers and nonsmokers.

Recovery of BAL fluid did not differ between smokers and nonsmokers. The total cell count in BAL fluid from smokers was nearly double the cell count in nonsmokers (*p* < 0.01). This was mainly due to higher numbers of macrophages and neutrophils. We found no significant differences in the absolute counts of lymphocytes and eosinophils in BAL samples between smokers and nonsmokers (Table 2).

### Gene expression of CYPs and FMOs

Expression of *CYP1A2*, *CYP2C8*, *CYP2C18*, *CYP2C19, CYP2D6, CYP3A7, CYP4A11, FMO1*, and *FMO3* was below the limit of detection (LOD) in all investigated samples (data not shown).

### Transcript profiling in BAL cells

**Figure 1:**
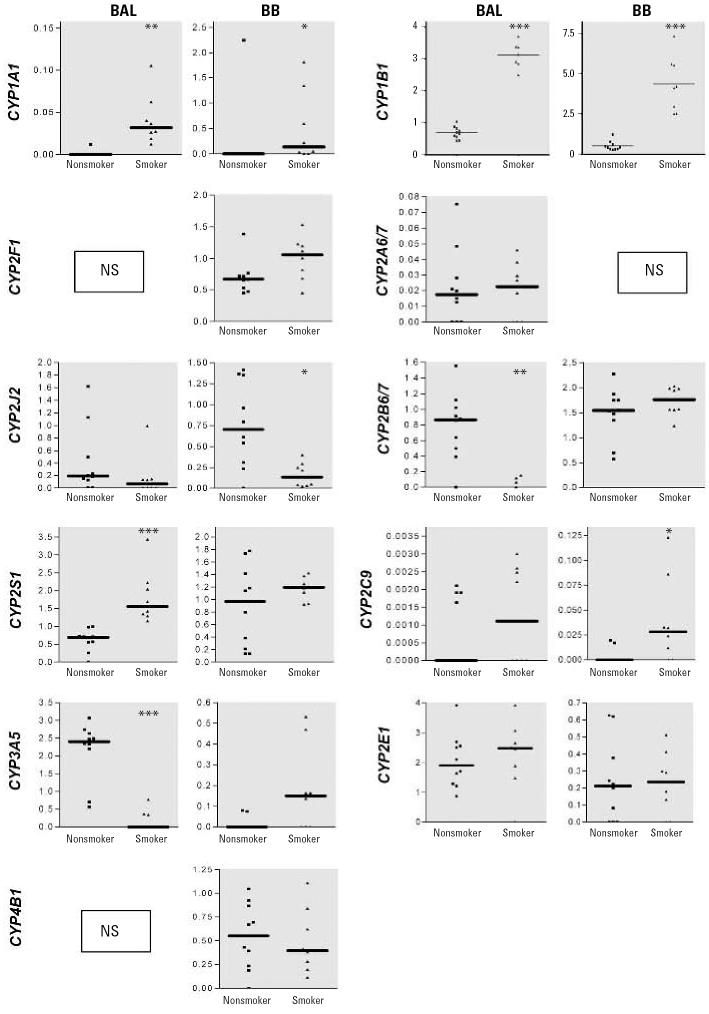
Gene expression of various CYPs in BAL cells and BB material obtained from nonsmokers (*n* = 10) and smokers (*n* = 8). NS, not significant. Data are represented as plots from individual volunteers, with solid lines indicating medians. Results are presented as the ratio of the gene of interest/cyclophilin; only signficantly altered genes are shown. *p < 0.05. **p < 0.01. ***p < 0.001.

Expression of *CYP1A1* (*p* < 0.01), *CYP1B1* (*p* < 0.001), and *CYP2S1* (*p* < 0.001) was higher in BAL cells from smokers compared with those of nonsmokers (Figure 1), whereas expression of *CYP2B6/7* (*p* < 0.01) and *CYP3A5* (*p* < 0.001) was lower; expression of *CYP2A6/7*, *CYP2E1*, *CYP2C9*, *CYP2J2*, and *FMO5* was not altered. *CYP2F1* transcripts were detected in 3 of 10 nonsmokers and in 3 of 8 smokers, but the amount was too small for semiquantitative analysis (data not shown). Similarly, *CYP4B1* was detected in 5 smokers and 1 nonsmoker, and *CYP3A4* was detected in 2 nonsmokers but was < LOD in smokers (data not shown).

### Transcript profiling in BBs

In smokers, expression of *CYP1A1* (*p* < 0.05), *CYP1B1* (*p* < 0.001), and *CYP2C9* (*p* < 0.05) was increased (*p* < 0.05) compared with non-smokers, and there was a trend toward increased expression of *CYP3A5* (*p* = 0.0545; Figure 1); however, the expression of *CYP2J2* was significantly (*p* < 0.05) repressed. Expression of *CYP2B6/7*, *CYP2E1*, *CYP2F1*, *CYP2S1*, *CYP4B1*, and *FMO5* was similar to that of nonsmokers. *CYP2A6/7* transcripts were detected in 2 of 10 nonsmokers and 3 of 8 smokers (data not shown). *CYP3A4* was < LOD in all BBs investigated.

### *Gene expression of* EPHX1, GST*s*, *and* UGT*s*

**Figure 2:**
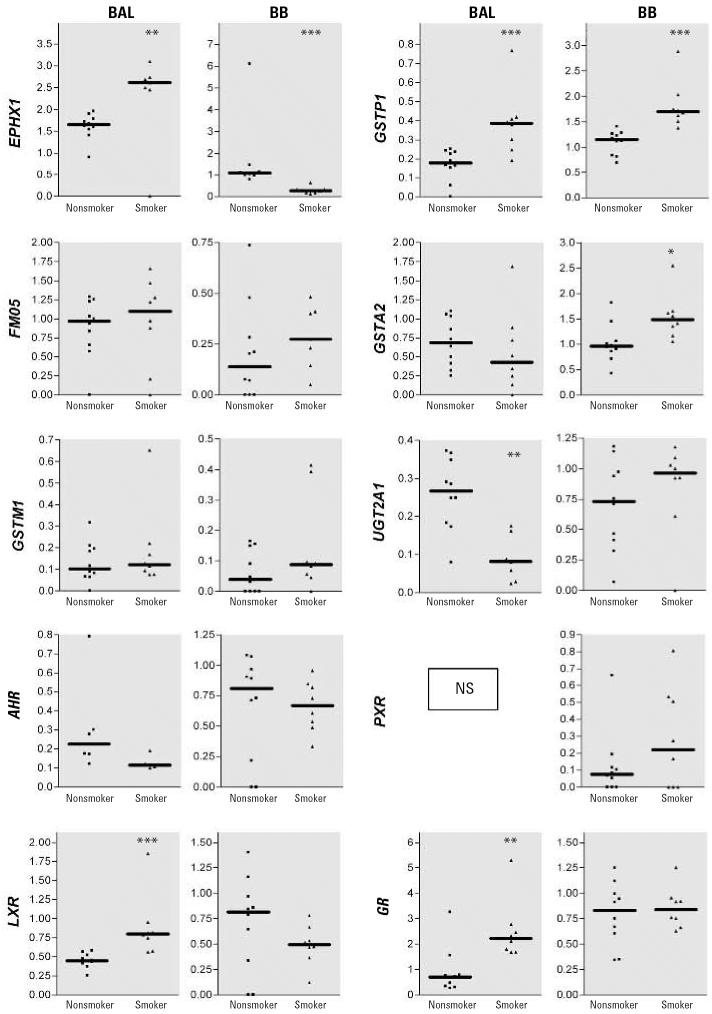
Gene expression of *FMO5*, various phase II enzymes, and key regulatory transcription factors in BAL cells and BB material obtained from nonsmokers (*n* = 10) and smokers (*n* = 8). NS, not significant. Data are represented as plots from individual volunteers, with solid lines indicating medians. Results are presented as the ratio of the gene of interest/cyclophilin; only significantly altered genes are shown. *p < 0.05. **p < 0.01. ***p = < 0.001.

In BAL cells of smokers, gene expression of *EPHX1* and *GSTP1* was significantly higher that that of nonsmokers, whereas expression of the *UGT2A1* gene was significantly lower (*p* < 0.001) (Figure 2). Expression of *GSTA2* and *GSTM1* was not different between the study groups (Figure 2).

Further, in BBs taken from smokers, expression of *EPHX1* was significantly lower (*p* < 0.001), whereas the expression of *GSTA2* and *GSTP1* were up to triple that in non-smokers. Expression of *GSTM1* and *UGT2A1* was not significantly different in BBs of the two groups (Figure 2).

### Gene expression of nuclear receptors

Gene expression of *LXR* (*p* < 0.001) and *GR* (*p* < 0.01) in BAL cells of smokers (Figure 2) was up to three times that of nonsmokers, whereas expression of the cytosolic *AHR* and *PXR* was similar. The expression of *AHR*, *LXR*, *PXR* and *GR* was not different in BBs of both study groups. Expression of *CAR* was < LOD in all samples studied.

### Enzyme activity of CYP1A1 and CYP1B1

**Figure 3:**
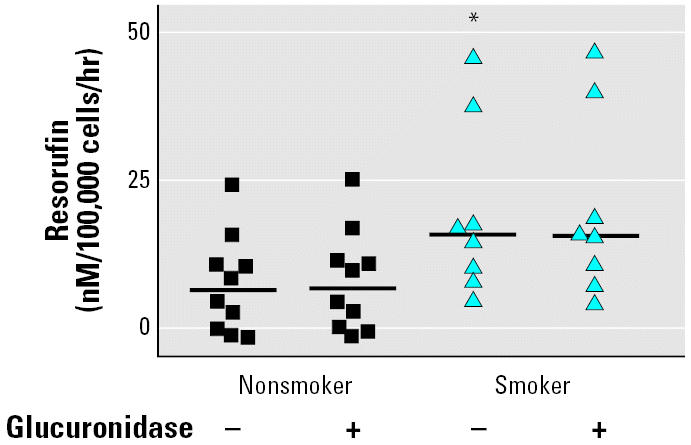
Metabolism of 7-ethoxyresorufin in BAL cells obtained from nonsmokers (*n* = 10) and smokers (*n* = 8) with and without glucuronidase treatment. Data represent mean ± SE of the individual incubation experiments, with approximately 100,000 cells per experiment. *p < 0.05.

7-Ethoxyresorufin served as substrate for *CYP1A1* and *CYP1B1*. In BAL cells derived from smokers, *EROD* activity was up to 3-fold that of nonsmokers (*p* < 0.05; Figure 3), but glucuronides were < LOD, as determined by β-glucuronidase treatment.

## Discussion

In this study we aimed to investigate the regulation of gene expression of major human pulmonary xenobiotic metabolizing enzymes as well as regulatory nuclear transcription factors in smoking and nonsmoking healthy volunteers. Differences were observed when we compared BAL cells and BBs of smokers and nonsmokers. Our findings provide new insight into lung tissue–specific responses to tobacco smoke as detailed below.

### Effects of tobacco smoke on pulmonary xenobiotic metabolizing enzymes

Lung tissue and cell types within the lung differ in their capacity to oxidize xenobiotics (Hecht 1999) because CYPs are not uniformly expressed (Ding and Karminsky 2003). As the majority of airborne toxicants enter the body through the respiratory tract, the pulmonary epithelium is exposed to high concentrations before systemic circulation, which, in turn, requires an effective local defense system. Our findings of a simultaneous induction of *CYP1A1* and *CYP1B1* in different lung compartments after exposure to tobacco smoke demonstrate up-regulation of the pulmonary enzyme system and agree well with other reports (McLemore et al. 1990). Regulation of *CYP1A1* and *CYP1B1* is not fully understood, but transcriptional activation by the AHR constitutes a major mechanism. Upon translocation into the nucleus, the AHR forms a heterodimer with its nuclear counterpart ARNT (AHR nuclear translocator) and drives gene expression of the *AHR*-responsible gene family, including *CYP1A1 and CYP1B1* (Borlak and Thum 2001). Thus, the strong induction of *CYP1A1* and *CYP1B1* can be explained, at least in part, in terms of AHR-mediated transcriptional activation. Although we did not observe changes of the *AHR* at the gene expression level, Martey et al. (2005) recently reported that cigarette smoke extract led to an activation of the *AHR* in cultured human lung fibroblasts based on induced nuclear translocation of the *AHR* (Martey et al. 2005). The observed up-regulation of *AHR*-responsive genes in lung tissue of smokers is likely mediated by the activation of *AHR*. The present study does, however, contradict the findings of Anttila et al. (1991), who did not find *CYP1A1* expression in alveolar macrophages, regardless of smoking status. This may be due to different experimental approaches because Anttila et al. employed immunohistochemistry, whereas we used a sensitive RT-PCR method determine *CYP1A1* and *CYP1B1* expression and a fluorometric assay to demonstrate increased activity of the coded enzymes. Indeed, Willey et al. (1996) were initially unable to detect *CYP1A1* expression in alveolar macrophages, but upon application of more sensitive techniques these investigator were able to detect *CYP1A1* gene expression (Willey et al. 1997).

CYP2A enzymes metabolize a variety of carcinogens including 4-(methylnitrosamino)-1-(3-pyridyl)-1-butanone (NNK), which can lead to the development of lung cancer (Koskela et al. 1999). In the present study, we detected expression of *CYP2A6/7* in both alveolar macrophages and bronchial biopsy material, but we did not find any difference in regard to smoking status. This contrasts the finding of Crawford et al. (1998), who demonstrated repression of *CYP2A6/7* in human bronchial epithelial cells of smokers. The somewhat high interindividual variation of *CYP2A6/7* gene expression observed in the present study and in the study of Crawford et al. (1998) make comparison of the data difficult.

We also observed significant induction of *CYP2S1* in BAL cells of smokers. Expression of *CYP2S1* in lung tissue has been previously reported (Rylander et al. 2001), but regulation by cigarette smoke has not been determined so far. One study (Rivera et al. 2002) demonstrated inducibility of *CYP2S1* in mouse lungs after systemic administration of dioxins. The observed induction of *CYP1A1* and *CYP2S1* in bronchoalveolar macrophages and bronchial biopsies of smokers is of importance because of their contribution to the metabolic activation of tobacco smoke components, namely polycyclic aromatic hydrocarbons, which can lead to the development of lung cancer (Georgiadis et al. 2005).

CYP2B6/7 and CYP3A5 play an important role in the metabolic activation of NNK and of benzo[*a*]pyrene (Code et al. 1997; Hecht 1999), but transcript levels and enzyme activity were repressed in BAL cells derived from smokers. Previously, Hukkanen et al. (2003) demonstrated decreased *CYP3A5* expression in alveolar macrophages of smokers. We now extend the findings of Hukkanen et al. (2003) to BBs and report a trend toward increased *CYP3A5* expression in smokers (*p* < 0.0545), which could result in local metabolic activation of carcinogens. Notably, the CYP3A family of monooxygenases plays a key role in pulmonary drug metabolism. Inhaled drugs, such as salmeterol, tiotropium, theophylline, or glucocorticoids (e.g., budesonide, prednisone), with an established role in chronic obstructive pulmonary disease or asthma treatment (Global Initiative for Asthma 2005; Global Initiative for Chronic Obstructive Lung Disease 2005) are substrates for CYP3A isoforms (Jonsson et al. 1995; Zevin and Benowitz 1999). Therefore, modulation of CYP3A enzyme activity in smokers may require dose adjustment for some inhaled drugs.

Furthermore, the CYP epoxygenases CYP2C and CYP2J2 are key players in the metabolism of arachidonic acid, resulting in the production of epoxyeicosatrienoic acids, some of which affect vascular and bronchial smooth muscle tone (Fisslthaler et al. 1999; Thum and Borlak 2004; Zeldin et al. 1995). In the present study, we observed reduced expression of *CYP2J2* in BBs of smokers, whereas *CYP2C9* was significantly induced in this lung compartment. This shift in the expression of the CYP epoxygenase gene might alter production of epoxy fatty acids, some of which are considered to be signalling molecules affecting vascular- and smooth muscle cell tone. Undoubtedly, future studies are needed to determine the regulation and the role of epoxyeicosatrienoic acids in smokers.

In addition, genetic variability in the coding of various *CYP* genes may affect enzyme activity (reviewed by Daly et al. 1994; Ingelman-Sundberg 2001). Likewise, changes in the expression of *CYP* transcripts may influence an individual’s capacity to convert different precarcinogenic compounds into their ultimate carcinogens; therefore, these *CYP* transcripts are of major importance for an individual’s susceptibility of developing chemically induced cancer. Certain CYP mono-oxygenases have an established role in an activation of precarcinogens to ultimate carcinogens of inhaled tobacco smoke toxicants (e.g., CYP1A1, CYP1A2, CYP1B1, CYP2E1, CYP3A4). The simultaneous induction of *CYP1A1* and *CYP1B1* in both types of lung cells obtained from BAL and pulmonary epithelial cells after smoking cigarettes, as found in the present study, may therefore enhance an individual susceptibility for chemically induced lung cancer.

In addition, expression of *EPHX1* was increased in BAL cells of smokers, but decreased in BBs. This is important because in the absence of *EPHX1* the tobacco smoke carcinogen benzo[*a*]pyrene is primarily metabolized to the noncarcinogenic 7,8-benzo-phenolic product (Shou et al. 1994). Bartsch et al. (1992) observed a significant increase in the activity of pulmonary EPHX1 within smokers, but EPHX1 activity was determined in preparations of parenchymal lung tissue, which consists of many different cell types. Besides, the lung tissue specimens were taken from patients with either lung cancer or non-neoplastic lung diseases. Thus, a direct comparison between the present study and that of Bartsch et al. (1992) cannot be made. Our results demonstrate that enzyme activity of EPHX1 can be expressed differently in two lung tissue compartments.

We also observed induction of the carcinogen-metabolizing enzymes *GSTP1* in BAL cells and BBs and *GSTA2* in BBs from smokers. This is likely an adaptive response to chronic exposure to tobacco smoke components. GSTP1 overexpression inhibited cytotoxic effects of cigarette smoke extract on human fibroblast-derived cells and depletion of GSTP1-induced apoptosis in lung fibroblasts (Ishii et al. 2001, 2003). Moreover, Crawford et al. (2000) showed that mRNA levels of GSTs expressed by bronchial epithelial cells from patients with bronchogenic carcinoma are significantly lower compared with subjects without carcinoma. Thus, increased expression of *GSTP1* in healthy smokers might protect against accumulation of carcinogens.

### Effects on nuclear receptors

*GR* and *LXR* were significantly up-regulated in BAL cells, but no clear correlation between CYP mono-oxygenases and nuclear receptor gene expression was observed. However, the up-regulation of *GR* fits well to the increased expression of *CYP2C9* because GR plays an important role in transcriptional regulation of the *CYP2C9* gene (Gerbal-Chaloin et al. 2002). CYP regulation is, in most cases, not dependent only on a specific nuclear factor, but requires a complex network of interacting factors (Waxman 1999).

### Study limitations

We did not recruit for a sex-balanced study group; for example, 8 of 10 were females in the nonsmoking group, whereas 7 of 8 were males in the smoking group. Nonetheless, when gene expression was compared between men and women, we found no significant differences. Furthermore, within different lung tissue compartments, we observed opposite effects in expression of certain genes (i.e., *CYP3A5*, *EPHX1*) for smokers, but these were independent of the sex. Although drug administration before the bronchoscopic procedures was strictly controlled, we cannot rule out minor effects of the drugs used (e.g., midazolam, lidocain, atropine) on gene expression within lung tissue. Notably, the same drugs were given to both smokers and nonsmokers, usually ≤15 min before the bronchoscopic procedures. This short interval makes major effects on gene expression unlikely.

## Conclusions

We observed profound effects of tobacco smoke exposure in BBs and BAL cells of volunteers and found *CYP1A1, CYP1B1,* and *GSTP1* to be up-regulated in BBs and alveolar macrophages of smokers, whereas others transcripts were differentially expressed and, in some cases, even oppositely regulated (i.e., *CYP3A5*, *EPHX1*). Differences in the expression of xenobiotic metabolizing enzymes may suggest local and tissue-specific susceptibility in metabolically activated toxicity.

## Figures and Tables

**Table 1:** Subject characteristics.

Subjects	Age	Sex	FEV_1_ (% predicted)	FEV_1_/FVC (%)	Cigarettes/day	Pack-years	Cotinine (ng/mL)	Interval (min)^a^
Nonsmokers (*n* = 10)	23	F	108.5	73.7	0	0	8.5	—
	36	F	113.7	85.8	0	0	13.5	—
	21	F	92.2	77.1	0	0	0	—
	24	F	101.2	78.8	0	0	0	—
	28	M	106.0	76.3	0	3.75	20.4	—
	24	F	123.5	86.7	0	0	2.3	—
	25	F	105.5	83.8	0	0	0	—
	26	M	98.6	81.7	0	0	0	—
	26	F	85.5	79.7	0	0	0	—
	23	F	106.0	83.7	0	0	0	—
Median (range)	24.5 (21–36)	—	105.8 (85.5–123.5)	80.7 (73.7–86.7)	0 (0)	0 (0–3.75)	0 (0–20.4)	—
Smokers (*n* = 8)	28	M	107.6	85.2	20	13.0	194.8	87
	30	M	98.1	75.8	20	3.0	175.8	137
	22	F	113.2	83.2	20	7.0	400.0	157
	30	M	95.4	73.5	20	13.0	241.4	88
	29	M	89.2	78.6	20	2.5	156.8	93
	27	M	108.2	74.6	25	15.0	310.0	136
	26	M	99.0	83.7	20	12.0	369.0	88
	45	M	77.2	75.3	20	31.0	102.8	70
Median (range)	28.5 (22–45)	—	98.6 (77.2–113.2)	77.2 (73.5–85.2)	20 (20–25)	12.5 (2.5–31.0)	218.1 (102–400)	90.5 (70–157)

Abbreviations: F, female; M, male.

a Time interval between smoking the last cigarette to bronchoscopy.

**Table 2:** Basic BAL fluid data given as median (range).

			Macrophages		Neutrophils		Lymphocytes		Eosinophils	
Subjects (*n*)	Recovery (mL)	Total cells (× 10^6^)	Percent of cells	No. (× 10^6^)	Percent of cells	No. (× 10^6^)	Percent of cells	No. (× 10^6^)	Percent of cells	No. (× 10^6^)
Nonsmokers (10)	77.5 (60–88)	5.9 (2.5–7.0)	90 (84–95)	5.2 (2.2–6.7)	2 (1–3)	0.1 (0.1–0.2)	7 (3–13)	0.3 (0.1–0.8)	0 (0–1)	0 (0–0.1)
Smokers (8)	76.5 (62–86)	9.3 (5.3–23.6)**	90 (85–92)	8.1 (4.9–21.2)**	4 (1–8)**	0.5 (0.1–1.4)**	4.5 (3–5)	0.4 (0.2–1.2)	0 (0–4)	0 (0–0.3)

** *p* < 0.01 compared with nonsmokers.

**Table 3:** Oligonucleotide primers and gene regulation in BAL cells and BB samples.

Accession no.	Gene	Forward primer (5′–3′)	Reverse primer (5′–3′)	Product length (bp)	BAL	BB
CYPs						
D01150	*CYP1A1*	TCACAGACACAGCCTGATTGAG	GATGGGTTGACCCATAGCTT	432	↑	↑
NM_000104.2	*CYP1B1*	GCAGAATTGGATCAGGTCGT	TGGTCAGGTCCTTGTTGATG	301	↑	↑
M38504	*CYP1A2*	TGGCTTCTACATCCCCAAGAAAT	TTCATGGTCAGCCCGTAGAT	308		
U22027	*CYP2A6/7*	GTGTGGACATGATGCCGT	AGGACTTGAGGCGGAAGT	1,151		
X13494	*CYP2B6/7*	CCATACACAGAGGCAGTCAT	GGTGTCAGATCGATGTCTTC	357	↓	
XM_050922	*CYP2C8*	GATCATGTAATTGGCAGACACA	CCTGCTGAGAAAGGCATGAAG	311		
XM_050918	*CYP2C9*	AGCTTGGAAAACACTGCAGT	CCTGCTGAGAAAGGCATGAAG	437		↑
NM_000772	*CYP2C18*	CTGTAACTGATATGTTTGGG	CCTGCTGAGAAAGGCATGAAG	431		
NM_000769	*CYP2C19*	GTAATCACTGCAGCTGACTTAC	CCTGCTGAGAAAGGCATGAAG	431		
M33189	*CYP2D6*	TGATGAGAACCTGCGCATAG	ACCGATGACAGGTTGGTGAT	332		
AF084225	*CYP2E1*	AGCACAACTCTGAGATATGG	ATAGTCACTGTACTTGAACT	365		
J02906	*CYP2F1*	ATGAACTTGCCGCACCGCGT	ACAGGCTCCACTTACGGTGC	283		
AF272142	*CYP2J2*	CCCACCAAACTCTCTTCAGC	CATTCTCTGCACCTCATGGA	389		↓
AF335278	*CYP2S1*	ACCCCAACATCTTCAAGCAC	TTCATCTGGTCTGCGTGGT	312	↑	
X12387	*CYP3A4*	CCAAGCTATGCTCTTCACCG	TCAGGCTCCACTTACGGTGC	323		
L26985	*CYP3A5*	TGTCCAGCAGAAACTGCAAA	TTGAAGAAGTCCTTGCGTGTC	472	↓	
NM 000765.1	*CYP3A7*	CTATGATACTGTGCTACAGT	TCAGGCTCCACTTACGGTCT	474		
AF208532	*CYP4A11*	CAAGTGACCTCCCTGCTCAT	CTGATCTCCCCAGAATCACC	280		
NM 000779.1	*CYP4B1*	TGACCATGTGCATCAAAGGAG	AAAGCCATTCTTGGAGCGCA	397		
Other drug-metabolizing enzymes						
NM_000120	*EPHX1*	TGATGAGGGAGAGCGGGCTAC	TCAGCAGGTCGTCCAGGGAG	227	↑	↓
NM_002021	*FMO1*	GCTGTTCGAGTCCTGAAAGG	GCCAAAGAAGACGGTCAGAG	234		
NM_006894	*FMO3*	GGCAGGGCTAGCATTTACAA	GATGTCCGGAACAAACCATT	301		
NM_001461	*FMO5*	CTCTCAGTTTCATATTGCCCAG	ACATTATTTCTCTTATCTCTCAGG	400		
NM_000846	*GSTA2*	GCCCAAGCTCCACTACTTCA	GCAAGCTTGGCATCTTTTTC	354		↑
NM_000561	*GSTM1*	TTCCCAATCTGCCCTACTTG	GGGCTCAAATATACGGTGGA	347	↑	↑
XM_040116	*GSTP1*	ACCTCCGCTGCAAATACATC	GGGAGGTTCACGTACTCAGG	313	↓	
XM_003547	UGT2A1	TTGGCCAATGGAAGGTAGTC	TCCTGAGACACCATGTGGAA	297		
Nuclear transcription factors						
NM_001621	*AHR*	CTGCCTTTCCCACAAGATGT	GAAATTCAGCTCGGTCTTCG	352		
NM_005122	*NR1I3*	GTCATGGCCAGTAGGGAAGA	GTCCGGATCAGCTCTTCTTG	350	↑	
M10901	*NR3C1*	GCTCTGGGGTGGAGATCATA	TCCTTCCCTCTTGACAATGG	300	↑	
U22662	*NR1H3*	TCAACCCCATCTTCGAGTTC	GGGGACAGAACAGTCATTCG	351		
AF061056	*NR1I2*	TTGTTCGGCATCACAGGTAG	GGGATCTGAGGGATTTCTCC	351		
Housekeeping gene						
NM_021130	Cyclophilin	CTTGCCATTCCTGGACCCAA	TTTCGTGCTCTGAGCACTGG	347		

Accession numbers from GenBank (2005). Arrows indicate up- or down-regulation of significantly affected genes.
